# A clinician’s guide for administration of high-concentration and facilitated subcutaneous immunoglobulin replacement therapy in patients with primary immunodeficiency diseases

**DOI:** 10.1186/s13223-022-00726-7

**Published:** 2022-09-30

**Authors:** Kristin Epland, Daniel Suez, Kenneth Paris

**Affiliations:** 1 Infectious Disease Associates, Minneapolis, MN USA; 2 Allergy, Asthma and Immunology Clinic, PA, Irving, TX USA; 3 Division of Pediatric Allergy and Immunology, LSU Health Sciences Center New Orleans and Children’s Hospital New Orleans, 200 Henry Clay Avenue, New Orleans, LA USA

**Keywords:** Immunoglobulin replacement therapy, Intravenous immunoglobulin, Subcutaneous immunoglobulin, Facilitated subcutaneous immunoglobulin, Primary immunodeficiency diseases, Practical guidance

## Abstract

Immunoglobulin replacement therapy is the standard-of-care treatment for patients with primary immunodeficiency diseases who have impaired antibody production and function. Clinicians and patients may consider intravenous immunoglobulin (IVIG) or subcutaneous immunoglobulin (SCIG) options, and each route may offer different benefits for the individual. IVIG requires fewer infusion sites and less frequent infusions than some formulations of SCIG. However, SCIG does not require venous access, is associated with fewer systemic adverse infusion reactions than IVIG, and can independently be self-administered at home. Importantly, tailoring treatment experiences to the needs of the individual may improve treatment adherence and quality of life for patients with primary immunodeficiency diseases who often rely on long-term or lifelong treatment. This review aims to educate United States (US) healthcare providers on the administration process of SCIG, with a focus on more concentrated formulations of SCIG and facilitated SCIG. It provides practical guidance on initiating, optimizing, and monitoring SCIG therapy. The advantages and disadvantages of the different treatment options are also presented for discussion between the patient and clinician.

## Introduction

There are over 456 distinct genetic mutations associated with primary immunodeficiency diseases (PIDD)/inborn errors of immunity [1] leading to impairments in various components of the immune system and resulting in chronic, recurrent, and potentially life-threatening infections [2, 3]. The estimated prevalence of PIDD in the US is 1/1200 [4] with frequent ongoing discoveries of novel genetic defects resulting in PIDD. Based on the International Union of Immunological Societies (IUIS) classification, 10 categories are currently used to group PIDD (Table 1) [1, 5, 6]. Several organizations provide online resources for the diagnosis of PIDD, including the Immune Deficiency Foundation [7], the American Academy of Allergy, Asthma, and Immunology [2], and the Clinical Immunology Society [8].

**Table 1:** Summary of PIDD Categories

Major category	Subcategory
1. Immunodeficiencies affecting cellular and humoral immunity	• Severe combined immunodeficiencies, defined by CD3 T cell lymphopenia • Combined immunodeficiencies generally less profound than severe combined immunodeficiency
2. Combined immunodeficiencies with associated or syndromic features	• Immunodeficiency with congenital thrombocytopenia • DNA repair defects other than those listed in major category 1 • Thymic defects with additional congenital anomalies • Immuno-osseous dysplasias • Hyper IgE syndromes • Dyskeratosis congenita, myelodysplasia, short telomeres • Defects of vitamin B12 and folate metabolism • Anhidrotic ectodermal dysplasia with immunodeficiency • Calcium channel defects • Others
3. Predominantly antibody deficiencies	• Hypogammaglobulinemia • X-linked (Bruton’s) agammaglobulinemia (severe reduction in all serum immunoglobulin isotypes with profoundly decreased or absent B cells) • Other antibody deficiencies • Severe reduction in at least 2 serum immunoglobulin isotypes with normal or low number of B cells, CVID phenotype • Severe reduction in serum IgG and IgA with normal/elevated IgM and normal numbers of B cells, hyper IgM • Isotype, light chain, or functional deficiencies with generally normal numbers of B cells
4. Diseases of immune dysregulation	• HLH and EBV susceptibility • Syndromes with autoimmunity and others
5. Congenital defects of phagocyte number or function or both	• Congenital neutropenias • Functional defects • Defects of respiratory burst (chronic granulomatous disease) • Other non-lymphoid defects
6. Defects in intrinsic and innate immunity	• Bacterial and parasitic infections • Mendelian susceptibility to mycobacterial disease and viral infection
7. Autoinflammatory disorders	• Recurrent inflammation • Systemic inflammation with urticarial rash • Sterile inflammation (skin/bone/joints) • Type 1 interferonopathies • Others
8. Complement deficiencies	• Susceptibility to infection • Disseminated neisserial infections • Recurrent pyogenic infections • SLE-like syndrome • Atypical hemolytic uremic syndrome • Others
9. Bone marrow failure	• Fanconi anemia • Dyskeratosis congenita, myelodysplasia, defective telomere maintenance
10. Phenocopies of PIDD	• Associated with somatic mutations • Associated with autoantibodies

Adapted from Picard C et al. [5], Bousfiha A et al. [6]

All categories of PIDD are FDA-approved indications for intravenous or subcutaneous immunoglobulin replacement therapy.

*CVID* common variable immunodeficiency, *EBV* Epstein-Barr virus, *FDA* Food and Drug Administration, *HLH* hemophagocytic lymphohistiocytosis, *IgA* immunoglobulin A, *IgE* immunoglobulin E, *IgG* immunoglobulin G, *IgM* immunoglobulin M, *PIDD* primary immunodeficiency diseases, *SLE* systemic lupus erythematosus

Immunoglobulin (IG) replacement therapy (IGRT) is the standard-of-care treatment for patients with PIDD who have impaired antibody production and function [9–11] and can be administered through the intravenous (IV) route (IVIG) or subcutaneous (SC) route (SCIG). The American Academy of Asthma, Allergy, and Immunology, together with the Primary Immune Deficiency Subcommittee, has developed 8 guiding principles regarding the safe, effective, and appropriate use of IGRT in patients with PIDD (Table 2) [11].

**Table 2:** Guiding principles for use of IGRT in patients with PIDD

Guiding principle	Guiding principle rationale
Indication of immunoglobulin therapy	IGRT is indicated for patients with PIDD characterized by absent or deficient antibody production; PIDD is an FDA-approved indication for IGRT, for which all currently available products are licensed
Diagnoses	A large number of PIDD diagnoses exist for which IGRT is indicated and recommended; many present with low total levels of IgG, but some present with a normal level and documented specific antibody deficiency
Frequency of IGRT treatment	Treatment is indicated as ongoing replacement therapy for PIDD; treatment should not be interrupted once a definitive diagnosis has been established
Dose	IVIG is indicated for patients with PIDD at a starting dose of 400–600 mg/kg every 3–4 wks; SCIG is generally used at a starting dose of 100–200 mg/kg/wk; SCIG dosing frequency is flexible; less frequent treatment or use of lower doses is not substantiated by clinical data
IgG trough levels	Baseline IgG levels should not be used as the sole criterion upon which to base treatment decisions and can be used in association with clinical and other patient-specific factors to guide IGRT dosing
Site of care	The decision to infuse IVIG in a hospital, hospital outpatient, community office, or home-based setting must be based on clinical characteristics of the patient; SCIG is administered primarily in a home-based setting
Route	Route of IGRT administration must be based on patient characteristics; throughout life, certain patients may be more appropriate for IV or SC therapy depending on many factors, and patients should have access to either route as needed
Product	IVIG/SCIG are not generic drugs and products are not interchangeable; a specific product needs to be matched to patient characteristics to ensure patient safety; a change of product should occur only with the active participation of the prescribing physician

Adapted from Perez EE et al. [11]

*FDA* Food and Drug Administration, *IG* immunoglobulin, *IgG* immunoglobulin G, *IGRT* immunoglobulin replacement therapy, *IVIG* intravenous immunoglobulin, *PIDD* primary immunodeficiency diseases, *SCIG* subcutaneous immunoglobulin

The first patient to receive IGRT for PIDD was a child with the earliest description of agammaglobulinemia [12]. This patient had experienced at least 19 instances of clinical sepsis within 4 years [12]. Bruton successfully treated this patient with monthly intervals of SCIG, leading to a year free of sepsis [12]. Widespread IGRT was initially administered intramuscularly, yet due to serious local side effects, was dose-limited and did not sufficiently raise serum immunoglobulin G (IgG) levels [13, 14]. The US Food and Drug Administration (FDA) approval of IVIG in 1979 offered an effective option for patients who met the indications for IGRT [13]. For decades, IVIG was the only IgG treatment modality available until SCIG was approved by the FDA in 2006 [13].

Compared with IVIG, SCIG therapy does not require venous access, may be less time-consuming, can be self-administered at home or administered in a healthcare setting, and is associated with fewer systemic adverse infusion reactions, primarily because the monthly dose is divided into smaller daily, twice weekly, weekly, or biweekly doses [15]. Generally, conventional SCIG (cSCIG) infusions require more frequent administration (usually ranging from daily to once every 2 weeks) and a larger number of infusion sites than IVIG [10]. There are a variety of options (Table 3) available for higher concentration (≥ 16.5%) cSCIG or facilitated formulations compared with IVIG products of 5% or 10% concentration. One therapy, facilitated SCIG (fSCIG), uses facilitated delivery via recombinant human hyaluronidase, which allows for longer treatment intervals similar to IVIG [11].

**Table 3:** Current US-available high-concentration and facilitated immunoglobulin products and their properties

Route	Product^a^	Dosage Form	Diluent	Osmolality (mOsm/kg)	Sodium	pH	IgA (mcg/mL)	Stabilizer or Regulator	Pathogen Inactivation/Removal^b^
SC	Cutaquig	16.5% solution	NA	310–380	< 30 mmol/L	5.0–5.5	≤ 600	Maltose	CEF, UF, CHROM, S/D, pH 4
	Cuvitru	20% solution	NA	208–290	None	4.6–5.1	80	Glycine	CEF, CHROM, NF, S/D
	Hizentra	20% liquid	NA	380	Trace, < 10 mmol/L	4.6–5.2	≤ 50	Proline	CEF, CHROM, pH 4.2, DF, NF, VF, OAF
	Hyqvia	10% liquid + hyaluronidase, human recombinant	NA	240–300	None added	4.6–5.1	37	Glycine	CEF, CHROM, S/D, pH 4, NF
	Xembify	20% solution	NA	280–404	None	4.1–4.8	Not defined	Glycine	CEF, CHROM, CAP, NF, DF, low Ph
Intramuscular	GamaSTAN	16.5% solution	NA	Not available	Not measured	4.1–4.8	Not measured	Glycine	CEF, CAP, CHROM, NF, low pH, DF
	GamaSTAN S/D^c^	15–18% liquid	NA	Not available	0.4–0.5%	6.4–7.2	Not measured	Glycine	CEF, S/D, UF

Adapted from Perez EE et al. [11], which also describes IVIG and lower-concentration SCIG options.

^a^Brand names and descriptions refer to products in the US and some other countries; product availability, specific composition, and other details regarding individual products vary in other countries. Refer to additional UpToDate topics on immunoglobulin therapy and product inserts for the indications and use of these products.

^b^Pathogen inactivation/removal using CEF, DF, UF, CAP, CHROM, Nano, double sequential nanofiltration, VF, S/D, Past, PEG, FP, or OAF.

^c^GamaSTAN S/D has been discontinued in the US.

*CAP* caprylate,* CEF* cold ethanol fractionation,* CHROM* chromatography,* DIF* dual inactivation and filtration,* DF* depth filtration,* FP* fraction precipitation,* IV* intravenous,* IVIG* intravenous immunoglobulin,* NA* not applicable,* Nano NF* nanofiltration,* OAF* octanoic acid fractionation,* past* pasteurization,* PEG* PEG precipitation,* S/D* solvent detergent,* SC* subcutaneous,* SCIG* subcutaneous immunoglobulin,* UF* ultrafiltration,* US* United States,* VF* virus filtration

Compared with less concentrated cSCIG products, those with higher concentrations allow for the infusion of a smaller volume of IG and reduced time spent on infusion [11]. Higher-concentration products are similarly tolerated (and in some studies preferred) by patients compared with lower-concentration and IVIG bioequivalents [16–18]. Clinically, lower-concentration products are not often used for PIDD. Several higher concentration cSCIG products are currently available in the US including Cutaquig (16.5% IgG) [19], Hizentra (20% IgG) [20], Cuvitru (20% IgG) [21], and Xembify (20% IgG) [22]. Another SCIG option is fSCIG, a dual-vial unit of IgG 10% and recombinant human hyaluronidase (rHuPH20) [23, 24]. The initial infusion of rHuPH20 increases the dispersion and absorption of immune globulin infusion 10% (human) by locally increasing the permeability of SC tissue via the temporary depolymerization of hyaluronan (a polysaccharide found in the extracellular matrix of connective tissue) [23]. This allows for SC administration of larger IgG infusion volumes and higher infusion rates relative to cSCIG [10, 25]. Due to differences in bioavailability, a prerequisite for FDA approval requires raising the IG dose by approximately 40% when switching from IVIG to cSCIG therapy for most of the commercially available SCIG preparations. Because these IGRT treatments may be used long term or often over the course of a lifetime, patients and prescribers need to consider infusion parameters (eg, route and site[s] of administration, frequency, and dose), administration setting, treatment tolerability, patient preference, and ability to self-inject and fit with patient lifestyle [26, 27]. Notably, home-based SCIG infusions are associated with improved health-related quality of life (HRQoL) in some patients with PIDD [28–33], and IGRT administration (SCIG or IVIG) at home is associated with lower direct healthcare costs than hospital administration [34, 35].

This review aims to educate US healthcare providers (HCPs) on the administration process of SCIG when used by patients with PIDD, with a focus on more concentrated formulations and fSCIG. It also provides practical guidance on initiating SCIG treatment, transitioning to home-based SCIG therapy, and optimizing and monitoring SCIG therapy. A broad range of patients with PIDD are candidates for SCIG therapy, and it is important for providers to engage patients who are willing and able to learn administration techniques with teaching assistance. To improve compliance with therapy, the advantages and disadvantages of SCIG therapy options need to be thoroughly discussed between the HCP and the patient and family in each case, tailoring the appropriate treatment to the patient’s needs and lifestyle. Thus, US HCPs can harness the benefits of SCIG to improve HRQoL and potentially improve patient outcomes.

## Initiating SCIG/fSCIG treatment for patients with PIDD

For patients with PIDD who transition to a new IGRT formulation, therapy is to be individualized, while taking into account that SCIG therapy is as effective with or without prior IVIG administration (ie, in IG-naïve patients) [15, 36–39]. In our clinical experience, attainment of steady-state IgG levels in patients with agammaglobulinemia may take longer if SCIG is initiated without prior IVIG. Patients usually initiate SCIG at a dose of 100–200 mg/kg of body weight each week [40], and dosing is subsequently adjusted according to serum IgG levels and clinical response (ie, frequency of infections) [41]. Clinical trials have utilized a dose ramp-up period with SCIG to transition patients to large volume SC infusion [42], although it is not often utilized in the real-world clinical setting and is not necessary for safety or efficacy reasons [43–45]. In practice, clinicians may find that ramping up the volume per infusion site is useful for patient comfort (ie, reduced pain or swelling) when initiating SC therapy. More frequent dosing of SCIG is necessary at treatment onset for patients with agammaglobulinemia or very low IgG levels to achieve therapeutic IgG serum levels more rapidly. Treating healthcare professionals should counsel patients that the SCIG infusion frequency may lead to more local adverse reactions. For IG-naïve patients, there are several strategies for initiating SCIG therapy [44, 46, 47]. These include initiation with a loading dose (SCIG or IVIG) for patients with very low IgG levels [44, 46, 47]. Some patients treated long-term with lower volumes of IG per site may be reluctant to try higher volumes compared with patients who started therapy with relatively higher infusion volumes per site.

While not yet addressed in guidelines, there has been a shift in dosing calculations from using actual body weight to ideal body weight as a marker of lean body mass, given appropriate patient monitoring and dose adjustments [48, 49]. This may be due to the use of less IG per patient for cost savings or because of drug shortages, and specialists are comfortable using ideal body weight to determine starting dose as long as there is flexibility to subsequently dose-adjust for desired IG levels and clinical response. Historically, a conversion factor for the transition from IVIG to SCIG has been used so that patients have the same level of IgG in their tissues from receiving SCIG as they would from IVIG over the course of IgG half-life [50]. Thus, for patients already receiving IVIG, the total monthly dose is multiplied by 1.37 for 16% IgG formulations or by 1.53 for 20% IgG formulations, and then is divided by the number of SCIG infusions administered per month [50]. Clinical trials in the US have used the area under the serum concentration–time curve (AUC) to determine SC- versus IV-administered IG bioavailability [50]. The pivotal fSCIG study following AUC analysis used a dose increase of 108% from IVIG to fSCIG on transition [23] to achieve a bioavailability of 93%, within the tolerance of 80–125% permitted by the FDA for bioequivalence, and therefore no conversion factor was needed. This compares to a suggested dose adjustment in the US of 137% from IVIG to cSCIG [51]. This more complicated conversion is rarely utilized in the real-world clinical setting.

Typically, in real-world situations in the European Union and the US, dosing is often 1:1 between IVIG and SCIG [52]. Trough IgG is used as a surrogate marker of adequate IgG replacement to evaluate IgG levels for patients on IVIG and for occasional measurements in patients on SCIG, and further adjustments are frequently based on clinical monitoring of infections. Comparing bioavailability by AUC and IgG trough levels in clinical practice is not straightforward, because a 1:1 switch from IVIG to SCIG leads to a 17% rise in trough IgG level [52]. Higher AUC-based dosing may improve infection-related and other patient-oriented outcomes [53], and several recent analyses showed that serum IgG levels are inversely correlated with annualized infection rates (Box 1) [54, 55]. In our opinion, acceptable IgG levels for a patient on IGRT would fall between 700–1600 mg/dL. Discrepancies between dosing regimens and pharmacokinetic parameters are frequently due to variations in each patient’s pharmacokinetics [41] and highlight the need for individualized treatment plans based on clinical response.

**Table:** 

Box 1 Serum IgG levels with IVIG, cSCIG, or fSCIG
IVIG results in a rapid increase in serum IgG levels, reaching peak serum concentration at approximately 15 min [14]. A subsequent steep decline occurs in serum IgG levels in the 48 h after infusion [14]
In contrast, IgG absorption is slower with cSCIG than IVIG, reaching peak serum concentration 2–4 days after infusion [14, 41]. Steady-state serum IgG levels with weekly cSCIG are 10–20% higher than IgG trough levels with the same total monthly IVIG dose [41]. Therefore, the overall IgG level with cSCIG is more consistent than IVIG, with less extreme peak and trough levels. This is thought to contribute to the lower incidence of systemic adverse events with cSCIG than IVIG, without compromising efficacy [32, 56, 57]; tolerability of cSCIG therapy is primarily due to the lower dose administered per IGRT session. Additionally, because the shorter dosing intervals with cSCIG eliminate low trough levels between infusions, “wear-off” or “trough” effects that are often experienced with IVIG can be minimized [14, 56]. Similarly, the peak serum IgG level after fSCIG infusion is not as sharp or immediate compared to IVIG [52]

*cSCIG* conventional subcutaneous immunoglobulin, *fSCIG* facilitated subcutaneous immunoglobulin, *IgG* immunoglobulin G, *IGRT* immunoglobulin replacement therapy, *IVIG* intravenous immunoglobulin

Transitioning patients from IVIG to SCIG therapy, or initiating IG-naïve patients with SCIG therapy, must be initially conducted in a specialized infusion facility under the care of an experienced medical provider or by a home infusion nurse. There are multiple protocols to guide therapy for the initiating healthcare professional [44, 46, 47]. Patients must demonstrate the ability to self-administer SCIG therapy prior to the authorization of self-home-infusion therapy, where tolerability of SCIG therapy can be further assessed. For SCIG and fSCIG, proper technique is emphasized in training. In addition, prior to authorizing home-infusion therapy, the home must be evaluated to ensure an adequate aseptic environment. This emphasizes the need for providers to determine early on patients that are suitable for initiating home therapy and to work closely with the patients and nursing team to ensure patient proficiency and comfort in treatment.

Depending on the needs of the patient, cSCIG may be administered as frequently as every day or weekly [41], to biweekly [58]; cSCIG products include 10% [50, 59], 16.5% [60], and 20% [39, 61] IgG formulations (Table 3). Monthly SCIG dosing is possible with fSCIG as it allows higher infusion and absorption volume with the addition of hyaluronidase [62]. The pivotal clinical trial of fSCIG demonstrated the ability to administer the total monthly IG dose into a single site at volumes up to 600 mL/site, and fSCIG also allows for flexible SCIG dosing by varying both the number of infusion sites and time between infusions [42]. This results in pharmacodynamics that are more similar to those observed after IVIG infusions, rather than other cSCIG formulations [11, 25, 62]. fSCIG is a convenient option for patients with a busy schedule or those who prefer less frequent infusions or require larger IgG doses. Alternatively, with a broad range of dosing options available for the 16.5% and 20% cSCIG formulations, patients report that the higher concentration and lower infusion volumes are both tolerable and effective despite the more frequent administration compared with IVIG or fSCIG [11, 41, 58, 63, 64]. The 16.5% and 20% cSCIG formulations are more optimal than the lower concentration 10% formulations, which are not typically used because they require larger volumes, multiple infusion sites, and longer infusion times.

Given the variety of IGRTs available and the necessity to individualize treatment for each patient, a need exists for the development of practitioner guidelines regarding how to transition from IVIG to SCIG while gradually reducing dosing to the equivalent previous IVIG dosing, and considering clinical outcome and trough IgG levels. Such guidelines detailing how to initiate and monitor the transition would certainly be helpful. Ultimately, each patient responds differently to treatment, and the treating healthcare provider needs to adjust the dosing to the individual for optimal efficacy.

## Transitioning to home therapy

A successful transition to home-based infusions and to self-administration (or administration by a family member or caregiver) of SCIG requires clinicians to carefully prepare patients’ understanding and expectations (Box 2). To safely administer SCIG in the home setting, the environment must be clean, and the necessary supplies laid out in an orderly manner on a clean surface. These include SCIG/vials, syringes, infusion pump, tubing, needles, pooling bag (fSCIG), transfer spikes (for cSCIG vials), alcohol wipes, tape or bandages, gloves, and a sharps container. Although rarely used, typical supplies also include an epinephrine injection, and/or diphenhydramine prescriptions for allergic reactions.

During initial training sessions, nurses can provide patients with additional guidance [65], including helping patients troubleshoot any infusion-related problems and adjust subsequent infusions when needed. The Immunoglobulin National Society (IgNS) provides a national database of Ig Certified Nurses (IgCNs) who are experienced and up-to-date in IGRT therapy and are required to pass a national certification exam and recertification every 3 years [66].

**Table:** 

Box 2 Literature to share with patients as anticipatory guidance for initiating SCIG/fSCIG therapy
**Immunoglobulin Replacement Therapy: One Size Doesn’t Fit All**
https://ipopi.org/wp-content/uploads/2017/07/WEB_IPOPI_oneSize.pdf
• Describes factors for patients with PIDD to consider and discuss with their healthcare provider when selecting an immunoglobulin replacement therapy
**Guide to Immunoglobulin Replacement Therapy for People Living With PIDD**
https://primaryimmune.org/sites/default/files/publications/IDF%20Guide%20to%20Ig%20Therapy.pdf
• Reviews SCIG regimens, including dosing, side effects, monitoring, and practical considerations
• Compares IVIG, SCIG, and fSCIG treatment options
• Includes a troubleshooting guide for SCIG administration
• Links to additional educational and support resources for patients and families
**SCIG Infusions: A Practical Guide for Patients**
https://www.idfa.org.au/wp-content/uploads/2020/09/IPOPI_PID-SCIG_Infusions.pdf
• A step-by-step infusion guide, including equipment set-up, infusion site selection and preparation, infusion administration and monitoring, and clean-up
**SCIG Therapy General Information**
https://www.allergy.org.au/images/pcc/ASCIA_PCC_SCIg_General_Information_2021.pdf
• Condensed information packet that includes diagrams, pictures, and a management guide for problems or reactions with SCIG infusion
• Links patients to checklists for SCIG infusions and equipment
• Provides guidance for maintaining treatment plans with travel plans
**Selecting SCIG Pumps and Needle Sets**
http://www.igliving.com/magazine/articles/IGL_2015-04_AR_Product-Guide-Selecting-SCIG-Pumps-and-Needle-Sets.pdf
• A short overview of different products and supplies patients can request for their long-term treatment

*fSCIG* facilitated subcutaneous immunoglobulin, *IVIG* intravenous immunoglobulin, *PIDD* primary immunodeficiency diseases, *SCIG* subcutaneous immunoglobulin

## Optimizing and monitoring SCIG therapy

Optimizing high-concentration SCIG therapy requires adjustments to infusion parameters or use of specific equipment with consideration of individual patient IgG levels, clinical response, and comfort/preference. Clinicians may find that monitoring the patient’s clinical response (ie, frequency of infections) is more useful than monitoring the patient’s IgG levels when adjusting the dose of SCIG. However, monitoring IgG levels is still recommended to prevent serious infections because studies of SCIG have shown increases in serum IgG levels are associated with low annual infection rates [30, 51, 57]. A meta-analysis of studies of weekly SCIG infusions showed increasing serum IgG levels were significantly associated with decreasing annual infection rates. There was no specific IgG level that was adequate across all patients, but an individual patient’s basal IgG level may be considered in dosing [55, 56] and, in our experience, patients with specific clinical situations such as bronchiectasis or risk of bronchiectasis may require higher serum IgG levels.

To improve adherence to SCIG, providers can partner with nurses to offer individualized education and support to patients, which can result in several benefits to the patient (Table 4) [67–69]. A nurse-led, patient-centered, and individualized SCIG home-infusion program was evaluated for successful transitions from IVIG to SCIG [70]. Among patients with immune-mediated neuromuscular disorders, who typically require higher IGRT dosage with SCIG than patients with PIDD, 89.5% and 78.9% successfully transitioned to SCIG from IVIG at 6 and 12 months, respectively [70]. In a real-world study, 88% of patients with PIDD successfully completed 4 infusions when initiating SCIG (20% IgG) therapy in a patient support program.

**Table 4:** Benefits of nursing interventions

Intervention	Possible benefit(s)
Patient education Use of training aids (eg, in relation to IgG administration technique)	• Increased patient empowerment • More effective partnerships between HCP and their patients • Improved likelihood that home-based treatment will be administered correctly
Telephone liaison	• Regular contact with the patient improves the likelihood that adverse events or suboptimal treatment efficacy will be managed correctly and in a timely fashion • Use of the telephone reduces the number of pharmacy (and potentially health care facility) visits the patient needs to make
Patient monitoring using standard assessment tools and questionnaires	• Increased likelihood of treatment regimens being adjusted as needed for optimal efficacy • Reassures the patient that they are receiving high-quality care • Potentially reduces the number of hospital visits that the patient needs to make
Recommendation for dose adjustment to provider	• Facilitation of timely adjustments to the patient’s treatment, to ensure optimal disease management

Adapted from Tichy et al. 2020

*IgG* immunoglobulin G, *HCP* healthcare provider

### Practical administration guidance for patients

Patients with PIDD can take practical steps to improve the SCIG administration process. The clinician can provide patients with anticipatory guidance regarding the rate of infusion and how it can be adjusted to their preference. Patients can proactively work with a specialty pharmacist to improve infusion rates by varying different components of their infusion equipment, including needle gauge, tubing flow rate, disposable flow-rate controllers, and pump type (ie, manual versus electronic). For examples of literature to share with patients, see Box 2.

### Patient guidance for managing infusion site reactions

Knowledge of the patient’s treatment goals and expectations is important for providers as patients must receive additional guidance for managing local infusion site reactions. This is particularly crucial in patients initiating fSCIG, as recent clinical trials highlighted infusion reactions as one the primary reasons for treatment discontinuation in a small number of patients [44, 45, 52]. Although the number of patients was small, these dropouts occurred in highly controlled studies, with specifically selected patient populations, thorough patient training, and frequent monitoring; thus, these findings warrant consideration for patient adherence with fSCIG [44, 45, 52]. fSCIG initiation may require additional training and monitoring due to much higher volumes infused over a shorter timespan [44, 45]. Maintaining an open conversation on treatment type and goals may be particularly important for patients who have struggled to tolerate SCIG and fSCIG infusions. For many patients, local swelling, redness, pain, or itching are commonly associated with SCIG [67, 68]. These infusion site reactions are often mild, typically resolve within hours after the infusion, and decrease in frequency and intensity with time [67, 71]. Patients need to track the dimensions of any local reaction that increases in size and be in contact with a provider or nurse to monitor for potential infection [71]. Suggested are listed in Box 3 [52, 68, 72] and this anticipatory guidance may improve treatment-plan adherence.

It is important to emphasize that while converting patients to SCIG therapy, the first 2 to 3 sessions are to be conducted in the provider’s office or by a home infusion nurse to ensure that the patient demonstrates the ability to perform all required SCIG infusion steps on their own and to assess the patient’s tolerability of SCIG therapy. Patients opting for SCIG therapy must be regularly followed and monitored by the provider and staff to ensure compliance and proper therapy. It is extremely important to have patients work closely with nursing/training staff to ensure they have the necessary tools and guidance to optimize treatment.

**Table:** 

Box 3 Approaches for mitigating local site reactions	
	• Using a different needle length (a longer needle may be needed to reach the subcutaneous tissue and avoid discomfort)
	• Using a different needle gauge (a narrower gauge may reduce pain during needle insertion while a broader gauge may decrease resistance, increase the infusion rate, and decrease the infusion time)
	• Using a different type of medical tape or bandage (to mitigate reactions to certain adhesives, paper or hypoallergenic tape may be needed)
	• Ensuring a dry needle is used (do not expose the skin to liquid that is on the needle)
	• Decreasing infusion volume per site • Increasing infusion time to decrease burning sensation
	• Using gentle massage or applying a warm/cold compress after infusion

## Benefits of SCIG treatment

Beyond the safety and efficacy of SCIG demonstrated in children, adults, and elderly patients, SCIG treatment offers other benefits to patients [73]. As illustrated in the previous section, many aspects of SCIG treatment can be individualized. This may be of particular advantage in patients with difficult venous access, including infants, very small children, and adults with compromised access. The flexibility of infusion parameters also includes various infusion intervals that can accommodate a range of schedules, such as parents’ work schedules or young adults’ college classes [74]. Pediatric patients who have depended on a caretaker or parent to administer infusions can continue therapy as they mature, eventually self-administering SCIG according to their individual schedules. Adaptability of treatment may further provide developmental benefits in pediatric patients. Recent studies of pediatric patients with PIDD have found association with fatigue and school absences, as well as increase in anxiety and depressive symptoms and impaired emotional and social functioning [75, 76]. These studies emphasized the importance of tailoring treatment to each patient’s needs, and an additional therapy option with flexibility in dosing regimen may offer a route to improving HRQoL for these patients [75, 76].The total monthly dose prescribed by the clinician can be divided according to the interval between infusions (eg, a total monthly SCIG dose of 800 mg/kg can be divided into 200 mg/kg per week). The typical infusion interval for fSCIG is every 3 to 4 weeks and is preferred by patients who desire the convenience of home-based SCIG infusions but with fewer injections and longer intervals [44].

Patients can also opt for manual push administration rather than infusion pumps [63]. For patients who prefer more frequent dosing, manual push can maintain good tolerability and similar trough IgG levels and infection rates compared with infusion pumps [77]. Among patients with PIDD, SCIG delivered by manual push resulted in more rapid infusions and was most frequently used for pediatric patients (< 2 years of age) [63]. Manual push may also be more practical in countries with decreased access to infusion pumps and could save 70% of administration cost compared with pump infusions [77].

Many patients prefer home treatment for its important advantages [31]. Overall treatment costs are reduced by removing the need for transportation, potentially an accompanying family member or caregiver, and trained medical professionals [34, 73]. Better general health, reduced impact on daily activities, and better social functioning contribute to the improved quality of life reported with home-based SCIG [28–30]. Patients also consider the flexible treatment schedules and not needing to travel as notable advantages [78] which may also provide more freedom by patients not being required to live close to an infusion center and having more ability to travel.

It is important to note that home-based SCIG infusions may not suit all patients [78]. Some patients are not comfortable assuming responsibility for their own infusion therapy, maintaining the required supplies in an aseptic environment at home, or addressing potential acute side effects without direct medical supervision. Side effects and infusion site reactions are of particular importance, as previous studies have shown these are major hurdles to long-term patient adherence and QoL with SCIG and fSCIG therapy [44, 45, 52]. The need for multiple monthly infusions may also conflict with some patients’ scheduling needs. Lastly, financial considerations should be carefully tailored for each patient. SCIG products cost more per gram than most IVIG products, but overall costs are dependent on the site-of-care and payor site-of-care guidelines (which have become more prevalent since 2015) [79, 80]. Hospital administration on average tends to be more expansive than in-home treatment, even after factoring in reimbursement for nursing hours [79, 80]. Thus, it is important for prescribing physicians to discern which patients would best be suited for each administration option and maintain an open conversation with patients to tailor treatment based on patient needs, preferences, and treatment history. It is also crucial to approach treatment as a caregiver team, with close engagement between patients, infusion-specialized nursing, and social services to optimize treatment as well as navigate any insurance concerns.

## Conclusions

SCIG therapy has many advantages for patients with PIDD who rely on long-term or lifelong IGRT. SCIG does not require venous access, is associated with fewer systemic adverse reactions than IVIG, and local infusion site reactions are typically mild, resolve on their own, and reduce in frequency with repeated infusions. Patients in all age groups and their caregivers can benefit from the convenience of often self-administered home-based infusions, which can be individualized, empower a patient to manage their own treatment, and improve their quality of life. Still, IVIG requires fewer infusion sites and less frequent infusions than SCIG and therefore may better suit some patients. Clinicians must discuss the advantages and disadvantages of the different IGRT options with their patients and provide practical guidance for the treatment that best matches the needs of the patient.

## Data Availability

Not applicable.

## Abbreviations

cSCIG: Conventional subcutaneous immunoglobulin
FDA: Food and Drug Administration
fSCIG: Facilitated subcutaneous immunoglobulin
HCP: Healthcare provider
IG: Immunoglobulin
IgG: Immunoglobulin G
IGRT: Immunoglobulin replacement therapy
IV: Intravenous
IVIG: Intravenous immunoglobulin
PIDD: Primary immunodeficiency diseases
SC: Subcutaneous
SCIG: Subcutaneous immunoglobulin
US: United States
